# COMMUNITY MOBILITY IN OLDER ADULTS RECOVERING FROM DISABILITY IN DISINVESTED NEIGHBORHOODS: A QUALITATIVE STUDY

**DOI:** 10.1093/geroni/igae098.0074

**Published:** 2024-12-31

**Authors:** Jason Falvey, Lindsey Mathis, Jasmine Cooper-Williams

**Affiliations:** University of Maryland School of Medicine, Baltimore, Maryland, United States; University of Maryland Baltimore, Baltimore, Maryland, United States; University of Maryland Baltimore, Baltimore, Maryland, United States

## Abstract

In socioeconomically disadvantaged areas, crumbling infrastructure and perceptions of limited safety can reduce opportunities for older adults to safely get around outside and engage meaningfully in social activities. This study seeks to understand how older adults in disinvested communities meaningfully recover community mobility after disabling injuries, and evaluates how recent payment reforms have challenged individualized rehabilitation delivery in home and outpatient settings. We recruited 13 adults over the age of 55, residing in neighborhoods with an Area Deprivation Index (ADI) of ≥9/10. Participants engaged in one-hour semi-structured interviews, coded by two independent researchers. The reliability between coders was verified using a kappa statistic. Five critical themes describe these mobility experiences: 1) exacerbating impact of chronic diseases and physical limitations on community mobility 2) environmental and infrastructural barriers restricting access to community resources; 3) influence of perceived safety and violent crime on participation in outdoor activities; 4) essential role of social support networks in enhancing mobility and 5) power of individual and community resilience in mitigating mobility challenges. One participant noted: “I never know when they going to start shooting and stuff. So when it start getting dark, I get up pretty early and get out early, try to get my stuff done [before dark].” –a vivid illustration of modifications in mobility forced by the fear of gun violence, complicating the recovery process Our study suggests new care pathways are needed to enhance the support for older adults navigating the challenges of recovery within socioeconomically disadvantaged neighborhoods.

